# Identification and Characterization of Novel CFTR Potentiators

**DOI:** 10.3389/fphar.2018.01221

**Published:** 2018-10-26

**Authors:** Maarten Gees, Sara Musch, Steven Van der Plas, Anne-Sophie Wesse, Ann Vandevelde, Katleen Verdonck, Oscar Mammoliti, Tzyh-Chang Hwang, Kathleen Sonck, Pieter Stouten, Andrew M. Swensen, Mia Jans, Jan Van der Schueren, Luc Nelles, Martin Andrews, Katja Conrath

**Affiliations:** ^1^Galapagos NV, Mechelen, Belgium; ^2^Department of Medical Pharmacology and Physiology, University of Missouri, Columbia, MO, United States; ^3^AbbVie, North Chicago, IL, United States

**Keywords:** CF, CFTR, potentiator, bronchial epithelial cells, electrophysiology

## Abstract

There is still a high unmet need for the treatment of most patients with cystic fibrosis (CF). The identification and development of new Cystic Fibrosis Transmembrane conductance Regulator (CFTR) modulators is necessary to achieve higher clinical benefit in patients. In this report we describe the characterization of novel potentiators. From a small screening campaign on F508del CFTR, hits were developed leading to the identification of pre-clinical candidates GLPG1837 and GLPG2451, each derived from a distinct chemical series. Both drug candidates enhance WT CFTR activity as well as low temperature or corrector rescued F508del CFTR, and are able to improve channel activity on a series of Class III, IV CFTR mutants. The observed activities in YFP halide assays translated well to primary cells derived from CF lungs when measured using Trans-epithelial clamp circuit (TECC). Both potentiators improve F508del CFTR channel opening in a similar manner, increasing the open time and reducing the closed time of the channel. When evaluating the potentiators in a chronic setting on corrected F508del CFTR, no reduction of channel activity in presence of potentiator was observed. The current work identifies and characterizes novel CFTR potentiators GLPG1837 and GLPG2451, which may offer new therapeutic options for CF patients.

## Introduction

Cystic fibrosis (CF) is the most common genetic (autosomal recessive) disease in Caucasians, with an estimated 80,000 CF diagnosed cases worldwide (Lubamba et al., 2012; Hanrahan et al., 2013; Merk and Schubert-Zsilavecz, 2013; De Boeck et al., 2014). Current strategies for treatment of CF patients can be broadly divided into two main categories: agents that target downstream effects of Cystic Fibrosis Transmembrane conductance Regulator (CFTR) dysfunction (i.e., symptomatic treatment) and agents that target the root cause of the disease, i.e., CFTR modulators which address the absent or dysfunctional CFTR in epithelial membranes. The treatments in the latter category are further classified as either correctors, which increase the levels of CFTR present on the cell surface, or potentiators which enhance the function of CFTR channels, There are three approved CFTR modulator treatments available for CF patients, namely the potentiator Ivacaftor (Kalydeco^®^) or VX770, the corrector Lumacaftor or VX809 and the corrector Tezacaftor or VX661, The Ivacaftor/Lumacaftor combination therapy (Orkambi^®^) or the Ivacaftor/Tezacaftor combination therapy (Symedeko^®^) are available for the treatment of patients homozygous for the F508del CFTR mutation. However, these treatments result in only minor benefit to these patients (Wainwright et al., 2015; Taylor-Cousar et al., 2017) and thus there is a demand for improved combinations to further improve clinical benefit for CF patients with the F508del mutation.

Potentiators can only function if CFTR is already present at the cell membrane, and work by increasing the opening probability (Po) of CFTR (Moran, 2010). VX770 is a potentiator which improves the channel opening of CFTR mutants with gating or conductance defects (class III and IV mutations, respectively) such as G551D (Van Goor et al., 2009; Yu et al., 2012; Jih and Hwang, 2013) and R117H (Yu et al., 2016). Overall, these two classes of mutations that could benefit from VX770 represent approximately 8–10% of the total patient population. Clinical trials indeed showed significant benefit in these patient populations with VX770 including an improvement of lung function by 10.6% (Ramsey et al., 2011; Hadida et al., 2014). The availability of additional potentiator molecules with improved potency, efficacy, and safety could provide further benefit to that provided by VX770.

Here we describe the characterization of two chemically distinct clinical candidate potentiators. This characterization was performed by evaluating their activity using higher throughput methodologies such as yellow fluorescent protein (YFP)-halide assays, low throughput patch clamp assays and more clinically relevant assays using primary cells derived from CF patients.

## Materials and Methods

### Cell Cultures

CFBe41o- cells were cultured in Eagle’s minimal essential medium (MEM) (Life Technologies) supplemented with 10% fetal bovine serum (FBS), 1% penicillin/streptomycin, 1% L-glutamine and 500 μg/ml hygromycin B. The cells were grown on culture flasks coated with 0.01% bovine serum albumin (BSA) (Sigma), 30 μg/ml Purecol (Nutacon), and 0.001% human fibronectin (Sigma). A CFBe41o- cell line stably expressing F508del CFTR harboring an HRP-tag in the 4th extracellular loop was obtained from Professor Gergely Lukacs (Department of Physiology, McGill University, Montreal, Canada) (Veit et al., 2012). Cells were grown in MEM supplemented with 10% FBS, 1% L-glutamine (Life Technologies), 10 mM HEPES (Life Technologies), 200 μg/ml geneticin (Life Technologies) and 3 μg/ml puromycin (Sigma) on culture flasks coated as for CFBe41o-. HEK293 cells were cultured in uncoated flasks using Dulbecco’s Modified Eagle Medium (DMEM) (Life Technologies) supplemented with 10% FBS and 1% penicillin/streptomycin. CHO cells were cultured in DMEM containing 10% FBS.

### Human Bronchial Epithelial (HBE) Cell Culture

Bronchial epithelial cells isolated from transplanted lungs from wild-type (WT), CF patients homozygous for the F508del mutation or heterozygous G551D/F508del or R334W/F508del patients, were obtained from McGill University (Montreal, Canada) and University of North Carolina (Chapel Hill, NC, United States). Cells were isolated from lungs obtained from donors undergoing a planned transplantation. These primary cells were cultured directly on type IV collagen-coated polycarbonate Transwell supports with a diameter of 6.5 mm and pore size of 0.4 μm (Costar, #3397) for 18–25 days in air liquid (ali) interface essentially as previously described (Fulcher et al., 2005).

### Yellow Fluorescent Protein (YFP) Halide Assay (CFBE, Low Temperature Corrected)

CFBe41o- cells were plated in black 384-well microplates at a density of 1,500 cells per well. After 24 h, cells were transduced with adenoviruses containing F508del CFTR and YFP (H148Q/I152L/) F47L using a multiplicity of infection of 30 infectious units per cell for each adenovirus. The next day, for the low temperature corrected assay, cells were incubated at 27°C for 24 h, for the chemically corrected assay cells were treated with corrector for 24 h at 37°C. On the day of analysis, cells were washed five times with Dulbecco’s Phosphate-Buffered Saline (DPBS) containing Ca^2+^ and Mg^2+^ (Life Technologies) using a Microplate Washer (BioTek). Then, cells were treated with 10 μM forskolin and the desired concentration of potentiator in a volume of 30 μl and the plates were incubated at room temperature for 10 min, a timepoint optimized in previous experiments showing good window (positive control/negative control > 2) and signal to background ratio. The YFP fluorescence was recorded for 2 min, starting immediately before addition of 30 μl NaI buffer (375 mM NaI, 7.5 mM KI, 1.76 mM KH_2_PO_4_, 10.1 mM Na_2_HPO_4_, 13.75 mM glucose) to the wells, using a FDSS/μCell (Hamamatsu) fluorescence reader with a 480 nm excitation filter and a 540 nm emission filter. The capacity of potentiators to increase CFTR channel function was expressed as 1-[fluorescence 36 s after NaI addition (F)/fluorescence before NaI addition (F0)], a timepoint optimized previously, resulting in an optimal signal window. Data was normalized using the formula: normalized response = 100^∗^ (absolute response – negative control response)/(VX770 response – negative response) as such, VX770 response corresponds to 100. For Dose-response experiments, data was fitted using a 4 parameter hill function of the form Response = Bottom + (Top-Bottom)/(1 + 10ˆ((LogEC50-concentration)^∗^HillSlope)) to determine EC_50_ values.

### YFP Halide Assay (HEK293)

HEK293 cells were transfected with 10–80 ng of plasmid encoding G551D, G178R, G1349D, S549N, R117H, R334W, or wild type CFTR and 20 ng of plasmid encoding YFP (H148Q/I152L/F47L) using jetPEI (Polyplus transfection). Directly after transfection, cells were seeded in black 96-well plates coated with Poly-D-lysine at a density of 70,000 cells per well. The next day, cells were incubated at 27°C for 24 h, for low temperature correction. When evaluating in a chronic setting, the potentiator concentration range was also added to the cells for 24 h. After this incubation period, cells were washed twice with DPBS with Ca^2+^ and Mg^2+^. Subsequently, cells were treated with 10 μM forskolin and the desired concentration of potentiator in a volume of 40 μl and incubated at room temperature for 10 min, a timepoint optimized in previous experiments resulting in a good window (positive control/negative control > 2) and signal to background ratio. YFP fluorescence was measured using an EnVision plate reader (PerkinElmer). The signal was recorded for 7 s, starting just before injection of 110 μl NaI buffer (137 mM NaI, 2.7 mM KI, 1.7 mM KH_2_PO_4_, 10.1 mM Na_2_HPO_4_, 5 mM D-glucose) into the well with a speed of 150 μl/s, resulting in a final volume of 150 μl. The excitation wavelength was 485 nm and the emission wavelength 530 nm. The capacity of potentiators to increase CFTR channel function was expressed as 1-[fluorescence 7 s after NaI addition (F)/fluorescence before NaI addition (F0)]. Data was normalized using the formula: normalized response = 100^∗^ (absolute response – negative control response)/(VX770 response – negative response) as such, VX770 response corresponds to 100. For Dose-response experiments, data was fitted using a 4 parameter hill function of the form Response = Bottom + (Top-Bottom)/(1 + 10ˆ((LogEC50-concentration)^∗^HillSlope)) to determine EC_50_ values.

### Cell Surface Expression Assay

CFBE41o^-^ TetON cells expressing HRP tagged F508del-CFTR (Veit et al., 2012) were seeded at a density of 4,000 cells per well in white 384-well plates (Greiner). Medium containing 500 ng/ml doxycycline was used to induce expression of the F508del-CFTR–HRP construct. After 3 days, cells were treated with corrector and/or potentiator compounds and transferred to an incubator at 33°C. On day 4, cells were washed 5 times with PBS containing Ca^2+^ and Mg^2+^ using a Bio-Tek plate washer and incubated with a chemiluminescent HRP substrate (SuperSignal West Pico Chemiluminescent Substrate, Thermo Scientific) for 15 min. Chemiluminescence was measured using an Envision plate reader (Perkin Elmer).

### TECC Experiments

Trans-epithelial clamp circuit (TECC) recordings were performed using the TECC instrument developed and sold by EP Design (Bertem, Belgium). During the recording, the epithelial cells were bathed in a NaCl-Ringer solution (120 mM NaCl, 20 mM HEPES, 1.2 mM CaCl_2_, 1.2 mM MgCl_2_, 0.8 mM KH_2_PO_4_, 0.8 mM K_2_HPO_4_, 5 mM glucose, pH 7.4) on both the basolateral (640 μl) and the apical side (160 μl) and kept at 37°C. Apical amiloride was used to inhibit the endogenous ENaC currents (100 μM) while forskolin (10 μM) was applied on both the apical and basolateral sides to stimulate CFTR. All triggers and compounds used during the experiment were first dissolved in DMSO to a 1000 X concentrated solution, just prior to treatment a 10 X stock was prepared in the NaCl-Ringer solution which was used for addition of the correct concentration of trigger and or compound during the experiment. When using the F508del CFTR mutant, VX809 (3 μM) or GLPG2222 (1 μM) corrector was added on the basolateral side for 24 h to partially rescue F508del CFTR before adding the potentiator. For chronic experiments, the potentiator range was also added to the cells for 24 h and reapplied in the NaCl-ringer buffer prior to the electrophysiological recording. Measurements were done during a 20 min timeframe with recordings every 2 min. The transepithelial potential (PD) and transepithelial resistance (*R*_t_) were measured in an open circuit and transformed to *I*_eq_ using Ohm’s law. The increase in *I*_eq_ (Δ *I*_eq_) was used as a measure for the increased CFTR activity. EC_50_ values were generated by measuring impact of different concentrations of compound on *I*_eq_ in primary cells. For this purpose each transwell was treated with a different compound concentration. CFTRInh-172 was used at 10 μM to assess the specificity of the response. Each compound concentration was tested in duplicate or triplicate and standard error of mean (SEM) was calculated except for R334W/F508del which was single replicate but had a large concentration range showing a clear concentration-responsive effect. Data was fitted using a 3 parameter hill function of the form Response = Bottom + (Top-Bottom)/(1 + 10ˆ((LogEC50-concentration))) to determine EC_50_ values.

### Patch-Clamp Electrophysiological Recording

A more complete description of the patch-clamp methodology can be found in a recent publication (Lin et al., 2016). Briefly, CHO (Chinese Hamster Ovary) cells were transiently transfected with pcDNA plasmids containing various CFTR constructs and pEGFP-C3. After transfection, cells were trypsinized and plated onto 35 mm tissue culture dishes dispersed with sterilized glass chips. Electrophysiological experiments were performed 3–7 days following transfection.

Patch-clamp experiments were performed at room temperature with an EPC-9 patch clamp amplifier (HEKA Instruments, Holliston, MA, United States). Recording microelectrodes were made from borosilicate capillary glass with a two-stage pipette puller (Narishige, Tokyo, Japan). The pipette tip was polished with a home-made microforge before use. The pipette solution contained (in mM) 140 NMDG (*N*-methyl-d-glucamine)-Cl (Fisher Biotec), 2 MgCl_2_ (Fisher Biotec, Perth, Australia), 5 CaCl_2_ (Fisher Biotec), and 10 HEPES (Fisher Biotec), pH 7.4, with NMDG (see Lin et al., 2016 for details). The pipette resistance when filled with the regular pipette solution was 3–5 MΩ. Once the tip of the pipette and the cell membrane established GΩ resistance, the electrode was quickly pulled away from the cell to form an inside-out patch, where the cytosolic side of the membrane was exposed to the perfusion solution. Cells were perfused with a bath solution having (in mM) 145 NaCl (Fisher Biotec), 5 KCl (Fisher Biotec), 2 MgCl_2_, 1 CaCl_2_, 5 glucose (Fisher Biotec), 5 HEPES, and 20 sucrose (Fisher Biotec), pH 7.4, with NaOH (Fisher Biotec). After establishing an inside-out configuration, the patch was perfused with a standard perfusion solution (i.e., intracellular solution) containing (in mM) 150 NMDG-Cl, 2 MgCl_2_, 10 EGTA (Fisher Biotec), and 8 Tris (Fisher Biotec), pH 7.4, with NMDG (see Lin et al., 2016 for details) containing the catalytic subunit of protein kinase A (PKA) and 2 mM ATP. Once the phosphorylation-dependent activation of CFTR reached a plateau, 3 μM GLPG1837 or 10 μM GLPG2451 was applied in the continuous presence of 2 mM ATP until a steady state was attained.

Microscopic kinetic analysis was performed with a program provided by Dr. László Csanády (Semmelweis University, Budapest, Hungary) (Csanády, 2000). Experiments were performed 4-5 times and averages and SEM were calculated. The resulting Po values and single-channel kinetic parameters were compared with paired *t*-test (Excel, Microsoft); *P* < 0.05 is considered statistically significant.

## Results

**FIGURE 1:**
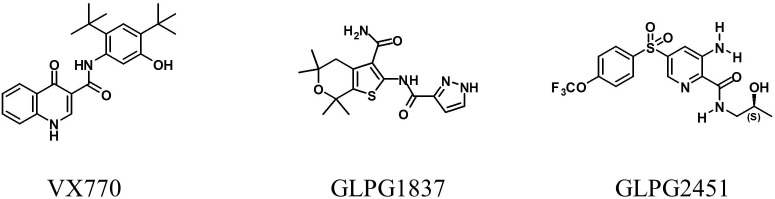
Structure of VX770, GLPG1837, and GLPG2451.

Small compound libraries, selected on the basis of molecular shape and electrostatic similarity to known potentiators and correctors, were screened for their ability to improve channel activity of low temperature rescued F508del CFTR using a YFP halide based assay. Several hits were identified including several series of structurally related compounds. Two of these series were selected for further medicinal chemistry efforts and optimization, resulting in the molecules GLPG1837 (described by Van der Plas et al., 2018) and GLPG2451. The chemical structures of both molecules and VX770 as comparator are represented in Figure 1.

**FIGURE 2:**
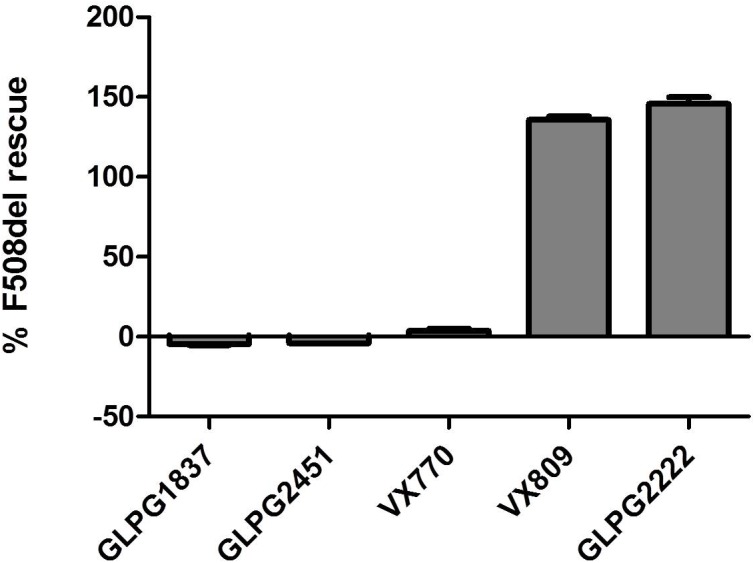
Cell Surface Expression assay: Rescue of HRP-tagged F508del CFTR in CFBe41O- cells was evaluated in presence of (from left to right), 10 μM GLPG1837, GLPG2451, VX770, VX809, or GLPG2222. Percent of cell surface rescue of F508del CFTR was calculated using an internal reference compound 15 (derived from same chemical series as GLPG2222). (*n* between 1 and 155 for different samples).

Both molecules effectively potentiate low temperature rescued F508del CFTR with an EC_50_ of 11.1 ± 3.6 nM (*n* = 10) and 3.5 ± 0.2 nM (*n* = 31) for GLPG2451 and GLPG1837 (a representation of the YFP quenching with time in the assay is represented in Supplementary Figure 1), respectively. Evaluation in a cell surface expression assay (Veit et al., 2014) shows that both molecules are not able to increase F508del CFTR levels at the plasma membrane and as such show no corrector activity (Figure 2).

### Characterization of Novel Potentiators on Class III Mutations Using YFP Halide Assay

**FIGURE 3:**
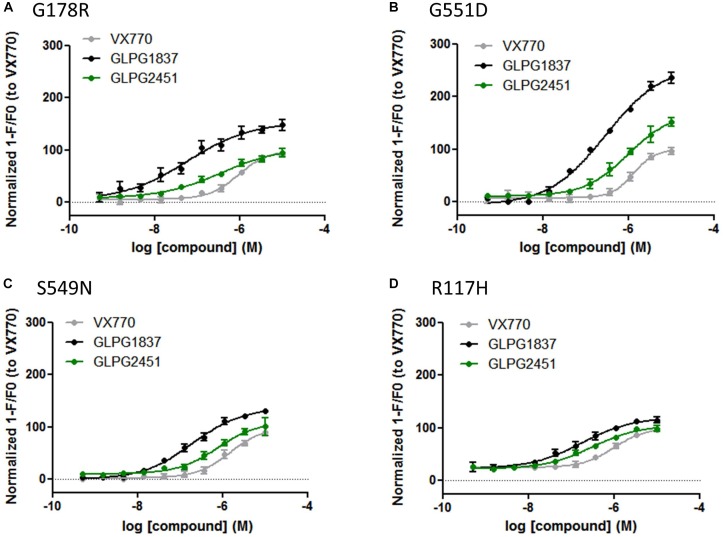
YFP-Halide assay on HEK cells expressing different CFTR mutants: Effect of a concentration range of GLPG1837, GLPG2451, or VX770 on **(A)** G178R CFTR, **(B)** G551D CFTR, **(C)** S549N CFTR, **(D)** R117H CFTR. 10 μM Forskolin was used for channel activation. The measured YFP fluorescence quenching was normalized to VX770 response (*n* between 2 and 8 for each concentration tested).

Both compounds were characterized for their ability to increase channel open probability of various gating defective CFTR mutants. Figure 3, represents a comparison of the potency and activity of VX770, GLPG1837, and GLPG2451 on various Class III CFTR mutations. A concentration-dependent increase in activity of G178R, S549N, G551D, and R117H CFTR was observed in the YFP halide assay. In this setting, GLPG1837 shows both a higher potency and a higher efficacy on all mutants tested when compared to VX770. The extent of the increased efficacy varies between the different mutations, 154% for G178R, 137% for S549N, 260% for G551D and 120% for R117H. The behavior of GLPG2451 is slightly different, being more potent on F508del CFTR when compared to VX770, but having similar potency on the other CFTR mutants evaluated. At saturation, the maximal ion channel activity is not as high as that seen for GLPG1837, but is similar to or higher than for VX770 (106% for G178R, 109% for S549N, 171% for G551D and 105% for R117H).

### Characterization Using Patient Derived Bronchial Epithelial Cells

**FIGURE 4:**
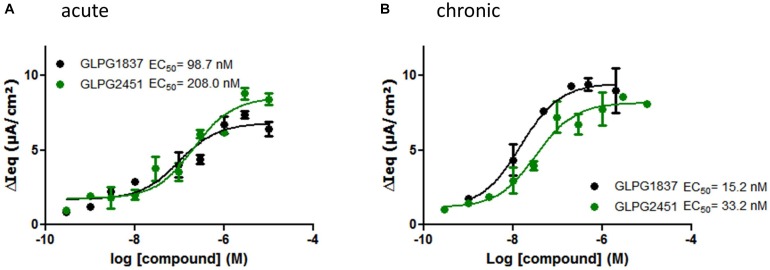
TECC assay on F508del/F508del HBEs: effect of a concentration range of GLPG1837, GLPG2451 on GLPG2222 corrected F508del CFTR. **(A)** Potentiators were added to the cells in “acute” setting, correctors were added 24 h prior to electrophysiological recording. **(B)** Corrector GLPG2222 and a concentration range of the potentiators were added to the cells 24 h prior to electrophysiological recording (“chronic” setting). Data represented is net current obtained from the combined FSK and potentiator treatment (tested in duplicate for each concentration, single F508del/F508del HBE donor shown).

Compound activity was then tested in a more physiologically relevant system, i.e., primary bronchial epithelial cells derived from CF patients. The effect on the function of VX809 (3 μM) corrected F508del/F508del CFTR was determined using an electrophysiological readout (TECC) and the data were compared to the data generated in the YFP halide assay. This showed the potency on VX809 corrected F508del/F508del HBEs to be lower (i.e., higher EC_50_) than on low temperature rescued F508del CFTR in a YFP halide assay [46.6 ± 14.2 nM (*n* = 6) vs. 3.5 ± 0.2 nM (*n* = 31) for GLPG1837 and 68.9 ± 25.7 nM (*n* = 4) compared to 11.1 ± 3.6 nM (*n* = 10) for GLPG2451 (representative curves in Figure 4A)]. This could be the result of differences in the cell background or assay sensitivity but could also result from subtle differences in the structures of low temperature corrected and chemically corrected F508del CFTR resulting in different compound potencies. Consistent with the latter interpretation, VX770 showed a similar potency in both assay formats implying it is not simply an assay difference and that these novel potentiators may act in a mechanistically different manner to VX770 (80.7 ± 21.7 nM (*n* = 7) (TECC) compared to 126.2 ± 10.9 nM (*n* = 56) (YFP halide assay), data not shown). To further understand this difference, a YFP halide assay was developed using a GLPG2222-like compound (1 μM) to chemically correct F508del CFTR which allows to determine the potency of the novel potentiators in a setting more similar to the HBE setting. In this case, the potency of both GLPG1837 and GLPG2451 corresponded closely to the potency obtained on chemically corrected F508del HBE cells (Supplementary Figure 2), suggesting that the potentiators can differentiate between chemical and temperature corrected F508del CFTR while this is not the case for VX770. Evaluation of low temperature corrected F508del HBE cells was not feasible.

The data in HBE cells were generated by simultaneous addition of potentiators and forskolin, to activate CFTR channels, a protocol referred to as “acute setting.” We also tested the compounds ability to potentiate F508del CFTR when incubated with the cells 24 h prior to the addition of forskolin, a setting called “chronic.” Under “chronic” conditions the potency of GLPG1837 and GLPG2451 improved ∼6 fold as compared to the “acute” setting (representative curves in Figure 4B).

As performed in the YFP halide assay, the effect of GLPG1837 and GLPG2451 on different gating defects was then investigated by TECC using G551D/F508del and R334W/F508del bronchial epithelial cells derived from CF patients.

**FIGURE 5:**
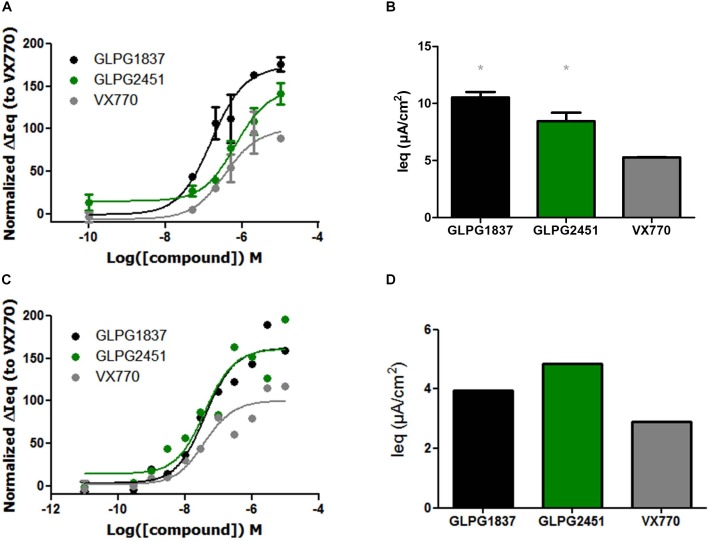
Effect of a concentration range or maximal efficacy at 3 μM of GLPG1837, GLPG2451, and VX770 on G551D/F508del **(A,B)** and R334W/F508del **(C,D)** primary human bronchial epithelial cells. (*n* = 2 for each tested concentration in G551D/F508del, *n* = 1 for each tested concentration in R334W/F508del), data in **(A,C)** was normalized to VX770. ^∗^ denotes a *P* < 0.05 compared to VX770 treatment. **(A)** Contains data for VX770 and GLPG1837 adapted with permission from Van der Plas et al. (2018). Copyright (2018) American Chemical Society.

On G551D/F508del cells, GLPG1837 had an EC_50_ value of 159 nM and an efficacy level of 173% of that of VX770. GLPG2451 had an EC_50_ value of 675 nM and an efficacy level of 147% of that of VX770 (Figures 5A,B). The R334W/F508del mutant has residual activity, but improved channel activity can be obtained by addition of a potentiator. In this setting a potency similar to that for corrected F508del CFTR was found (EC_50_ value of 40.7 and 40.3 nM for GLPG1837 and GLPG2451, respectively) again with a higher channel opening when compared to VX770 (162% for GLPG1837 and 161% for GLPG2451) (Figures 5C,D).

### GLPG1837 and GLPG2451 Activation of Wild Type (WT) CFTR

**FIGURE 6:**
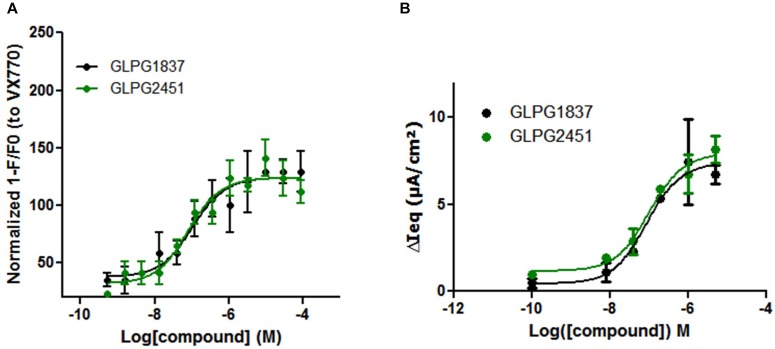
Effect of a concentration range of GLPG1837, GLPG2451 on WT CFTR activity. **(A)** Effect of GLPG1837, GLPG2451 on WT CFTR using a YFP halide assay in HEK293 cells. Data is normalized to the response observed for VX770 set at 100% and each condition was tested in triplicate. **(B)** Effect of GLPG1837 and GLPG2451 on WT CFTR expressed by primary human bronchial epithelial cells (tested in duplicate for each concentration, potentiator response is shown after FSK treatment).

Potentiators influence the open probability of CFTR and are expected to interact directly with CFTR. It has been demonstrated by Yu et al. (2012) that VX770 is able to improve Cl-current carried by WT CFTR. We investigated whether GLPG1837 and GLPG2451 could also improve WT CFTR channel activity. For this, a YFP halide assay was set up using WT CFTR and the assay was optimized to have maximal sensitivity by transfecting with low amounts of WT CFTR plasmid to avoid saturation of the YFP quenching signal. Upon activation of WT CFTR with 10 μM forskolin, GLPG1837 and GLPG2451 were able to increase the open probability with a potency of 103.5 and 79.7 nM, respectively, which is slightly weaker than their potency on F508del CFTR (Figure 6A). In primary cells derived from a WT CFTR donor, EC_50_ values of 88.0 and 102.5 nM after activation with 0.1 μM forskolin were found for GLPG1837 and GLPG2451, respectively, similar to the observations in the YFP halide assay (Figure 6B).

### GLPG1837 and GLPG2451 Improve CFTR Channel Function

**FIGURE 7:**
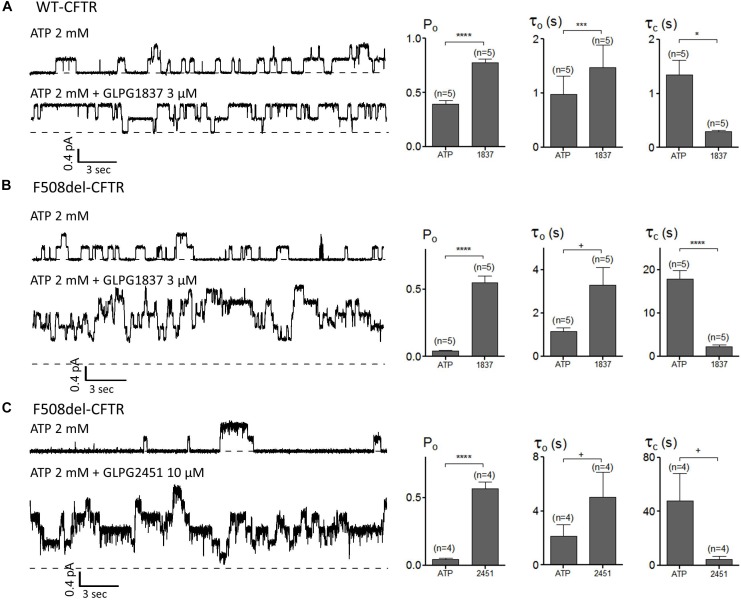
Patch clamp studies on excised patches using CHO cells showing the effect of GLPG1837 on **(A)** WT (Po, τ_o,_ and τ_c_), **(B)** F508del (Po, τ_o,_ and τ_c_) and of GLPG2451 on **(C)** F508del (Po, τ_o,_ and τ_c_) CFTR. The statistical analysis of single-channel kinetic parameters was described in Materials and Methods, every condition was tested at least 4 times. ^∗^*P* < 0.05; ^∗∗∗^*P* < 0.005; ^∗∗∗∗^*P* < 0.001; ^+^*P* > 0.05.

The impact of GLPG1837 and GLPG2451 on the CFTR channel activity was further investigated with the patch-clamp technique using WT and/or F508del CFTR. Inside-out patches were excised from cells transiently expressing WT or F508del CFTR, and application of the compounds was found to result in reversible potentiation of the activity of the channels when pre-activated with PKA and ATP. This activation can be seen immediately (within seconds) after addition of the compounds (an example using GLPG2451 is shown in Supplementary Figure 3). For WT CFTR, the Po before potentiator stimulation was 0.39 ± 0.04 (with τ = 0.97 ± 0.34 s and τ_c_ = 1.35 ± 0.27 s, *n* = 5), in the presence of 3 μM GLPG1837 the Po increased to 0.78 ± 0.04 (*n* = 5) with open time (τ_o_) and closed time (τ_c_) constants of 1.48 ± 0.41 s and 0.29 ± 0.03 s, respectively, a result comparable to those seen with VX770 (Jih and Hwang, 2013). For F508del, the Po was also dramatically increased to 0.55 ± 0.05 (*n* = 5) with τ_o_ = 3.29 ± 0.82 s and τ_c_ = 2.18 ± 0.52 s with GLPG1837 (Pre-potentiator Po was 0.04 ± 0.01 with τ_o_ = 1.16 ± 0.15 s and τ_c_ = 17.86 ± 1.90 s). Addition of GLPG2451 to F508del CFTR resulted in an increase in Po to 0.57 ± 0.05 (*n* = 4) with τ_o_ = 5.05 ± 1.82 s and τ_c_ = 4.44 ± 2.23 s (Pre-potentiator Po was 0.05 ± 0.01 with τ_o_ = 2.14 ± 0.86 s and τ_c_ = 47.88 ± 20.41 s) (Figure 7). When determining the channel opening probability impact on F508del CFTR, GLPG1837, and GLPG2451 both behave in a manner comparable to that of VX770, i.e., reducing the closed time and increasing the open time of the channel resulting in a net increase in Po to the comparable level. As there is always an uncertainty regarding the number of functional channels in the patch, the estimated Po should be considered as a maximal value.

### Are GLPG1837 and GLPG2451 Additive to Other Potentiators?

**FIGURE 8:**
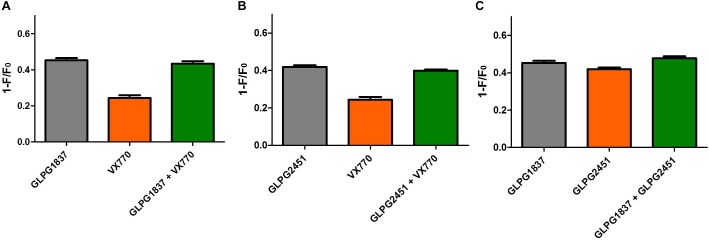
Evaluation of additivity of potentiators on G551D CFTR in a YFP-Halide assay using HEK cells. **(A)** Effect of 90 μM GLPG1837, 10 μM VX770 and combination of both using 1 μM forskolin for channel activiation. **(B)** Effect of 90 μM GLPG2451, 10 μM VX770 and combination of both using 1 μM forskolin for channel activation. **(C)** Effect of 90 μM GLPG1837, 90 μM GLPG2451 and combination of both using 1 μM forskolin for channel activiation. *n* = 9 for each condition.

A next question we wanted to address is whether the novel potentiators would be able to increase further the CFTR activity when combined with each other or VX770. Whilst using the YFP halide assay to look into additivity/synergy seemed straightforward, none of the combinations resulted in higher CFTR (WT of F508del) activity when compared to the single components (data not shown). The absence of any additional effect on CFTR activity could be due to both potentiators having the same binding site, as molecules are then competing to bind CFTR or due to the dynamic window of the YFP halide assay which might not allow us to see further increase in CFTR activity. Since the maximal effect on WT or F508del CFTR with different potentiators was similar, but different in the case of G551D, it was decided to evaluate potentiator effects on G551D CFTR using the YFP halide assay (Figure 8). The experiments showed that GLPG2451 and GLPG1837 failed to show any additive effect, suggesting that both compounds either bind in the same area or are hampering each other’s binding (Figure 8C). The two potentiators also did not show any additive effect to VX770 (Figures 8A,B). A possible limitation to this assay is that a Po of 1 could be reached and therefore no additivity would be observed. However, single channel patch clamp suggests that a Po of only 0.78 ± 0.04 is reached for WT CFTR and 0.55 ± 0.05 for F508del (Figure 7). While single channel studies on G551D CFTR were unsuccessful due to the low Po, it is highly unlikely that it would be reaching 1 when treated with either GLPG1837 or GLPG2451. Secondly, it could be that the signal in the assay itself reaches saturation, resulting in an apparent lack of additivity; we therefore performed similar experiments at a lower FSK concentration of 0.3 μM (data not shown). While these experiments resulted in a reduced assay window with higher variability, the data support a similar conclusion as presented with the higher forskolin concentration. In the case of GLPG1837, these data support the observations from Yeh et al. (2017) which suggested GLPG1837 and VX770 may share the same binding site.

### GLPG1837 and GLPG2451 Have a Greatly Reduced Detrimental Effect on Surface Expression of Corrected F508del CFTR After Chronic Exposure

**FIGURE 9:**
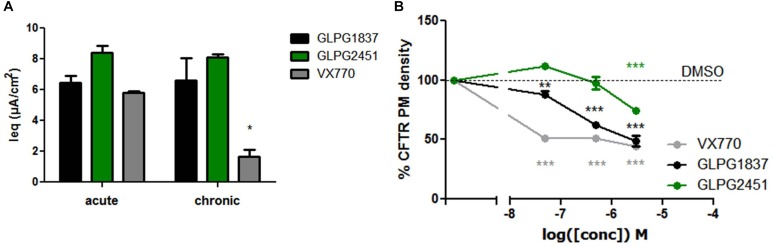
Treatment of F508del CFTR expressing cells corrected with VX809 or GLPG2222 with potentiator in acute or chronic setting. **(A)** Activity of F508del CFTR in primary HBE cells treated for 24 h with 3 μM corrector VX809 (for evaluation with VX770) or 0.15 μM GLPG2222 (in combination with GLPG1837 or GLPG2451). Potentiators were added at 10 μM together with the corrector (“chronic”) or just prior to the electrophysiological recording (“acute”). *N* = 3 for each condition, ^∗^ denotes a *P* < 0.05 compared to similar compound treatment in the acute condition. **(B)** Rescue of HRP-tagged F508del CFTR in CFBe41O- cells treated for 24 h with 10 μM VX809 in combination with a concentration range of VX770, GLPG1837, or GLPG2451. Data was normalized to cells treated with VX809 only. In black, results for GLPG1837 are shown, in green, results for GLPG2451 and in gray results for VX770 (*n* > 2 for each point). ^∗∗^*P* < 0.01; ^∗∗∗^*P* < 0.005 compared to the control condition without compound treatment.

Recently, several groups published data on the potential inhibitory effect of chronically exposed VX770 on VX809 corrected F508del CFTR leading to reduced amounts of corrected VX809 F508del CFTR on the plasma membrane (Cholon et al., 2014; Veit et al., 2014; Avramescu et al., 2017). The translation to clinical efficacy of these observations at the concentrations evaluated is improbable, however not all types of potentiators show this effect under similar conditions (Veit et al., 2014). We thus included this analysis here. Addition of 10 μM of either GLPG1837, GLPG2451, or VX770 to VX809 corrected F508del/F508del HBE cells in a “chronic” and “acute” setting shows a decrease in F508del CFTR channel activity using VX770 in “chronic” setting, but this negative drug–drug interaction was not observed for GLPG2451 and GLPG1837 in similar experimental conditions (Figure 9A). A concentration range of the three potentiators was also evaluated in the cell surface expression assay using F508del CFTR tagged with HRP. F508del CFTR was first partially rescued with VX809 and co-incubated with various concentrations of potentiators. After 24 h co-incubation, the amount of rescued F508del CFTR present at the plasma membrane was determined by measuring the horseradish peroxidase activity. Figure 9B shows concentration-dependence of these potentiators on surface expression of F508del CFTR. While VX770 reduces the level of cell surface expressed F508del CFTR at all concentrations tested, a higher concentration of GLPG2451 or GLPG1837 is required to see this detrimental effect. In addition, between GLPG2451 and GLPG1837, GLPG1837 appears to be slightly more potent in decreasing the effect of corrector VX809.

## Discussion

There are currently three approved CFTR modulator treatments available for cystic fibrosis patients, namely Ivacaftor, Orkambi and Symdeko. Of these, Ivacaftor (VX770) is a potentiator which improves the channel function for CFTR mutants with a gating or a conductance defect (Van Goor et al., 2009; Yu et al., 2012). In a clinical trial for patients carrying the G551D mutation, Ivacaftor improved the lung function by 10.6% (Ramsey et al., 2011). Orkambi and Symdeko on the other hand are combination therapies containing VX770 and VX809 or VX661, respectively, and are used for the treatment of patients homozygous for the F508del CFTR mutation. These later treatments show only minor benefit to the patients (Wainwright et al., 2015). Thus, there is a demand for the identification and development of novel correctors and potentiators, or more potent combinations of CFTR modulators for more effective therapy for CF patients with the F508del mutation.

This paper described the characterization of two chemically distinct potentiators GLPG1837 and GLPG2451 which have recently been identified (Van der Plas et al., 2018). Whilst other potentiators with interesting biological activities (absence of detrimental effect on the surface expression of corrected F508del CFTR and good efficacy on G551D CFTR) have recently been reported (Phuan et al., 2015; Park et al., 2016), these are not suitable for clinical development.

Both GLPG1837 and GLPG2451 are able to potentiate F508del, and some of the Class III and IV mutant CFTR. The pharmacological effects measured with YFP halide assays were found to correlate well to the results from more physiologically relevant assays using patient derived primary cells. In the case of F508del CFTR, the potency observed in the YFP halide assay on low temperature rescued CFTR was higher compared to chemical rescued F508del CFTR in both YFP halide and primary HBE cells. This difference was not observed for VX770 used in the same assay settings, suggesting that the novel potentiators may act on CFTR by a different binding mechanism. However, patch clamp data on F508del CFTR showed both GLPG1837 and GLPG2451 enhance the open probability by prolonging the open time and reducing the closed time, similar to that measured in a similar setting for VX770 (Jih and Hwang, 2013). The extent of activation of, e.g., G551D observed with GLPG1837 and GLPG2451 was higher compared to that seen with VX770 supporting a potentially different mechanism of action for the potentiators. Yeh et al. (2017) reported that GLPG1837 induced a 27.5-fold increase of macroscopic G551D CFTR current in comparison to a 10-fold increase by VX770 described in Jih and Hwang (2013). This increase in macroscopic current on G551D is in line with the observed higher channel activity observed in G551D/F508del bronchial epithelial cells in TECC.

We investigated whether both potentiators could act additively to each other or to other potentiators. On F508del CFTR, no further increase in CFTR was observed after addition of any second potentiator to GLPG1837 or GLPG2451. These data suggest that the potentiators cannot bind at the same time to F508del CFTR, or if they do, no further activation of the channel is possible. Yeh et al. (2017) suggested GLPG1837 and VX770 both bind to a similar epitope on CFTR using the patch clamp technique. Since for all potentiators a similar maximal signal is observed when using WT or F508del CFTR, use of these constructs does not allow for the assessment of whether they compete for a similar binding site.

On G551D CFTR, however, several of the potentiators yielded a different maximal quenching rate and therefore we investigated possible interactions between potentiators. GLPG1837 and GLPG2451 didn’t show any additive effect to each other nor to VX770, suggesting that the potentiators compete for the same binding site or binding of one hampers binding of the other.

All these data demonstrate that the interactions between potentiators and the channel are complex and additional experiments including structural as well as mechanistic studies would be needed to better understand the binding site and mode of action of these potentiators.

Recently, two groups published data on the potential inhibitory effect of chronically dosed VX770 on VX809 corrected F508del CFTR leading to reduced levels of VX809 corrected F508del CFTR at the plasma membrane (Cholon et al., 2014; Veit et al., 2014; Avramescu et al., 2017). The clinical impact of this observation remains, however, unknown. In this study; the impact of chronic incubation of GLPG1837, GLPG2451, or VX770 together with VX809 was assessed in two different types of assays. When adding a high concentration of potentiator (10 μM) together with VX809 in primary bronchial epithelial cells derived from a F508del/F508del homozygous patient, the detrimental effect of VX770 on VX809 rescued F508del CFTR was observed, comparable to the data presented previously by Cholon et al. (2014). In the same setting, both GLPG1837 and GLPG2451 did not show this effect. Similar effects of VX770 were noted when looking into the effect of the potentiators using a cell surface expression assay as described by Veit et al. (2014). In the same assay, GLPG1837 also showed some reduction of partial rescued F508del CFTR but with a concentration response curve shifted to the right when compared to VX770. GLPG2451 on the other hand showed a minor effect on F508del CFTR rescued protein only at the highest concentration evaluated. These data show that there is a difference in sensitivity toward the negative effect of potentiators on partial rescued F508del CFTR, depending on the assay format used. Overall, GLPG1837 and GLPG2451 reduce membrane density of VX809 rescued F508del CFTR to a lesser extent than VX770 but to a slightly higher extent compared to the potentiator P5 described by Veit et al. (2014).

Based on the more desirable properties of GLPG1837 and GLPG2451 as potentiators, further testing was performed to determine suitability for use in the clinic. GLPG1837 was evaluated in humans and was well tolerated in healthy volunteers, with single doses up to 2,000 mg and 14 days dosing up to 800 mg (Vanhoutte et al., 2015). Similarly, GLPG2451 was evaluated in humans and was well tolerated in healthy volunteers (Brearley et al., 2017). These data supported progression of GLPG1837 and GLPG2451 into Phase 2 studies. Two studies have been designed based on the *in vitro* characteristics of GLPG1837 on Class III CFTR mutants. GLPG1837 has been dosed in patients harboring a G551D or S1251N CFTR mutation. A phase 1b study evaluating the triple combination of GLPG2451, GLPG2222, and GLPG2737 in F508del CF patients is currently ongoing.

In summary, having additional therapeutic options available is of high interest to the CF community. Here, we have characterized two CFTR potentiators with distinct chemical structures GLPG1837 and GLPG2451, the hallmark of which is their higher efficacy for promoting Class III mutant channel gating in comparison to VX770. Having these molecules available to the CF field will enable a better understanding of the molecular mechanism of defects associated with different CFTR variants. Moreover, further testing and optimization of these may pave the way for the development of additional treatment regimens for patients with CF.

## Author Contributions

MG, SM, KC and JVdS conceptualized the data. MG, SM, SVdP, A-SW, KV, AV, KS, OM, T-CH, PS, AS, MJ, LN, and MA investigated the data. KC wrote the original draft. MG, SM, and MA wrote, reviewed, and edited the manuscript. MG, SM, and KC visualized the data.

## Conflict of Interest Statement

MG, SM, SVdP, A-SW, AV, KV, KS, PS, MJ, JVdS, LN, MA, and KC are employees of Galapagos and may own stock options in that company. AS was an employee of AbbVie. The remaining authors declare that the research was conducted in the absence of any commercial or financial relationships that could be construed as a potential conflict of interest.

## References

[B1] Avramescu R. G. Kai Y. Xu H. Bidaud-Meynard A. Schnúr A. Frenkiel S. (2017). Mutation-specific downregulation of CFTR2 variants by gating potentiators. *Hum. Mol. Genet.* 26 4873–4885. 10.1093/hmg/ddx367 29040544 PMC5886047

[B2] Brearley C. Gesson C. Kanters D. Cock P. Conrath K. Corveleyn S. (2017). *Safety, Tolerability and Pharmacokinetics of a Novel CFTR Potentiator GLPG2451 with and Without a Novel CFTR Corrector GLPG2222 in Healthy Volunteers*. Available at: http://www.glpg.com/docs/view/59fc5e55eaea7-en

[B3] Cholon D. M. Quinney N. L. Fulcher M. L. Esther C. R. Das J. Dokholyan N. V. (2014). Potentiator ivacaftor abrogates pharmacological correction of F508 CFTR in cystic fibrosis. *Sci. Transl. Med.* 6:246ra96. 10.1126/scitranslmed.3008680 25101886 PMC4272825

[B4] Csanády L. (2000). Rapid kinetic analysis of multichannel records by a simultaneous fit to all dwell-time histograms. *Biophys. J.* 78 785–799. 10.1016/S0006-3495(00)76636-7 10653791 PMC1300681

[B5] De Boeck K. Zolin A. Cuppens H. Olesen H. V. V. Viviani L. (2014). The relative frequency of CFTR mutation classes in European patients with cystic fibrosis. *J. Cyst. Fibros.* 13 403–409. 10.1016/j.jcf.2013.12.003 24440181

[B6] Fulcher M. L. Gabriel S. Burns K. A. Yankaskas J. R. Randell S. H. (2005). Well-differentiated human airway epithelial cell cultures. *Methods Mol. Med.* 107 183–206. 15492373 10.1385/1-59259-861-7:183

[B7] Hadida S. Van Goor F. Zhou J. Arumugam V. McCartney J. Hazlewood A. (2014). Discovery of N -(2,4-Di- tert -butyl-5-hydroxyphenyl)-4-oxo-1,4-dihydroquinoline-3-carboxamide (VX-770, Ivacaftor), a potent and orally bioavailable CFTR potentiator. *J. Med. Chem.* 57 9776–9795. 10.1021/jm5012808 25441013

[B8] Hanrahan J. W. Sampson H. M. Thomas D. Y. (2013). Novel pharmacological strategies to treat cystic fibrosis. *Trends Pharmacol. Sci.* 34 119–125. 10.1016/j.tips.2012.11.006 23380248

[B9] Jih K.-Y. Hwang T.-C. (2013). Vx-770 potentiates CFTR function by promoting decoupling between the gating cycle and ATP hydrolysis cycle. *Proc. Natl. Acad. Sci. U.S.A.* 110 4404–4409. 10.1073/pnas.1215982110 23440202 PMC3600496

[B10] Lin W.-Y. Sohma Y. Hwang T.-C. (2016). Synergistic potentiation of cystic fibrosis transmembrane conductance regulator gating by two chemically distinct potentiators, ivacaftor (VX-770) and 5-Nitro-2-(3-Phenylpropylamino) benzoate. *Mol. Pharmacol.* 90 275–285. 10.1124/mol.116.104570 27413118 PMC4998663

[B11] Lubamba B. Dhooghe B. Noel S. Leal T. (2012). Cystic fibrosis: insight into CFTR pathophysiology and pharmacotherapy. *Clin. Biochem.* 45 1132–1144. 10.1016/j.clinbiochem.2012.05.034 22698459

[B12] Merk D. Schubert-Zsilavecz M. (2013). Repairing mutated proteins–development of small molecules targeting defects in the cystic fibrosis transmembrane conductance regulator. *Expert Opin. Drug Discov.* 8 691–708. 10.1517/17460441.2013.788495 23574506

[B13] Moran O. (2010). Model of the cAMP activation of chloride transport by CFTR channel and the mechanism of potentiators. *J. Theor. Biol.* 262 73–79. 10.1016/j.jtbi.2009.08.032 19766125

[B14] Park J. Khloya P. Seo Y. Kumar S. Lee H. K. Jeon D.-K. (2016). Potentiation of ΔF508- and G551D-CFTR-mediated Cl- current by novel hydroxypyrazolines. *PLoS One* 11:e0149131. 10.1371/journal.pone.0149131 26863533 PMC4749168

[B15] Phuan P.-W. Veit G. Tan J. A. Finkbeiner W. E. Lukacs G. L. Verkman A. S. (2015). Potentiators of defective F508-CFTR gating that do not interfere with corrector action. *Mol. Pharmacol.* 88 791–799. 10.1124/mol.115.099689 26245207 PMC4576684

[B16] Ramsey B. W. Davies J. McElvaney N. G. Tullis E. Bell S. C. Døevínek P. (2011). A CFTR potentiator in patients with cystic fibrosis and the G551D mutation. *N. Engl. J. Med.* 365 1663–1672. 10.1056/NEJMoa1105185 22047557 PMC3230303

[B17] Taylor-Cousar J. L. Munck A. McKone E. F. van der Ent C. K. Moeller A. Simard C. (2017). Tezacaftor–ivacaftor in patients with cystic fibrosis homozygous for phe508del. *N. Engl. J. Med.* 377 2013–2023. 10.1056/NEJMoa1709846 29099344

[B18] Van der Plas S. E. Kelgtermans H. De Munck T. Martina S. L. X. Dropsit S. Quinton E. (2018). Discovery of *N*-(3-Carbamoyl-5,5,7,7-tetramethyl-5,7-dihydro-4*H*-thieno[2,3-c]pyran-2-yl)-l*H*-pyrazole-5-carboxamide (GLPG1837), a novel potentiator which can open class iii mutant cystic fibrosis transmembrane conductance regulator (CFTR) channels to a high. *J. Med. Chem.* 61 1425–1435. 10.1021/acs.jmedchem.7b01288 29148763

[B19] Van Goor F. Hadida S. Grootenhuis P. D. J. Burton B. Cao D. Neuberger T. (2009). Rescue of CF airway epithelial cell function *in vitro* by a CFTR potentiator, VX-770. *Proc. Natl. Acad. Sci. U.S.A.* 106 18825–18830. 10.1073/pnas.0904709106 19846789 PMC2773991

[B20] Vanhoutte F. Gouy M. Haazen W. Kanters D. De Beckker G. Geller D. (2015). “Safety, tolerability and pharmacokinetics of a novel CFTR potentiator GLPG1837 in healthy volunteers,” in *Poster Presented at North American Cystic Fibrosis Conference (NACFS)*, Phoenix, AZ.

[B21] Veit G. Avramescu R. G. Perdomo D. Phuan P.-W. Bagdany M. Apaja P. M. (2014). Some gating potentiators, including VX-770, diminish F508-CFTR functional expression. *Sci. Transl. Med* 6:246ra97. 10.1126/scitranslmed.3008889 25101887 PMC4467693

[B22] Veit G. Bossard F. Goepp J. Verkman A. S. Galietta L. J. V. Hanrahan J. W. (2012). Proinflammatory cytokine secretion is suppressed by TMEM16A or CFTR channel activity in human cystic fibrosis bronchial epithelia. *Mol. Biol. Cell* 23 4188–4202. 10.1091/mbc.e12-06-0424 22973054 PMC3484098

[B23] Wainwright C. E. Elborn J. S. Ramsey B. W. Marigowda G. Huang X. Cipolli M. (2015). Lumacaftor–Ivacaftor in patients with cystic fibrosis homozygous for Phe508del CFTR. *N. Engl. J. Med.* 373 220–231. 10.1056/NEJMoa1409547 25981758 PMC4764353

[B24] Yeh H.-I. Sohma Y. Conrath K. Hwang T.-C. (2017). A common mechanism for CFTR potentiators. *J. Gen. Physiol.* 149 1105–1118. 10.1085/jgp.201711886 29079713 PMC5715911

[B25] Yu H. Burton B. Huang C.-J. Worley J. Cao D. Johnson J. P. (2012). Ivacaftor potentiation of multiple CFTR channels with gating mutations. *J. Cyst. Fibros.* 11 237–245. 10.1016/j.jcf.2011.12.005 22293084

[B26] Yu Y.-C. Sohma Y. Hwang T.-C. (2016). On the mechanism of gating defects caused by the R117H mutation in cystic fibrosis transmembrane conductance regulator. *J. Physiol.* 594 3227–3244. 10.1113/JP271723 26846474 PMC4908022

