# Supplementary material for: Identification and Characterization of Novel CFTR Potentiators
Source: Front Pharmacol. 2018 Oct 26;9:1221. doi: 10.3389/fphar.2018.01221 (PMC6212544; doi:10.3389/fphar.2018.01221)
Supplement: Supplementary file 1 [file Data_Sheet_1.PDF]

Supplementary Material

Identification and Characterization of Novel CFTR Potentiators

Maarten Gees, Sara Musch, Steven Van der Plas, Anne-Sophie Wesse, Ann Vandevelde, Katleen Verdonck, Oscar Mammoliti, Tzyh-Chang Hwang, Kathleen Sonck, Pieter Stouten, Andrew M. Swensen, Mia Jans, Jan Van der Schueren, Luc Nelles, Martin Andrews, Katja Conrath*

*** Correspondence:** Corresponding Author: katja.conrath@glpg.com

1 Supplementary Figures

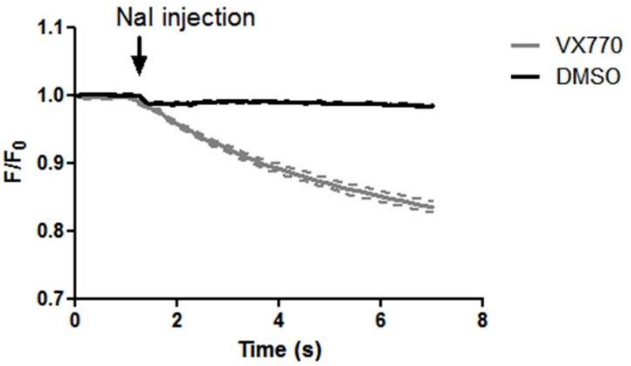

Supplementary Figure 1.

YFP-Halide assay using HEK293 cells over-expressing F508del CFTR: Example timecourse comparing DMSO treated cells with VX770 treated cells (average and SEM are plotted with n = 8 for each timepoint treated with VX770 and 20 for each DMSO treated timepoint)

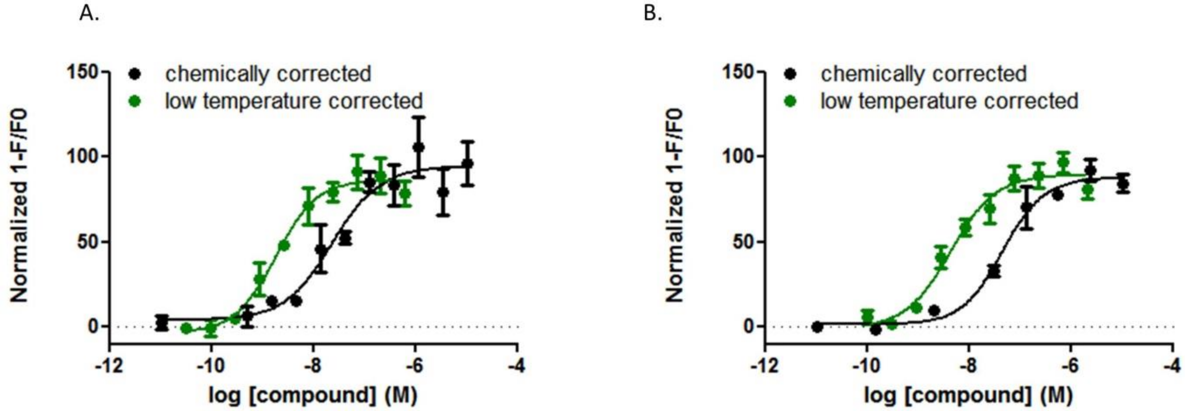

Supplementary Figure 2.

YFP-Halide assay using CFBe41o- cells: Potentiator effect after chemical correction or low temperature correction of F508del CFTR using potentiator GLPG1837 **(A)** or GLPG2451 **(B)**. 10µM Forskolin was used for channel activation. Example curves run in duplicate or triplicate at each concentration tested.

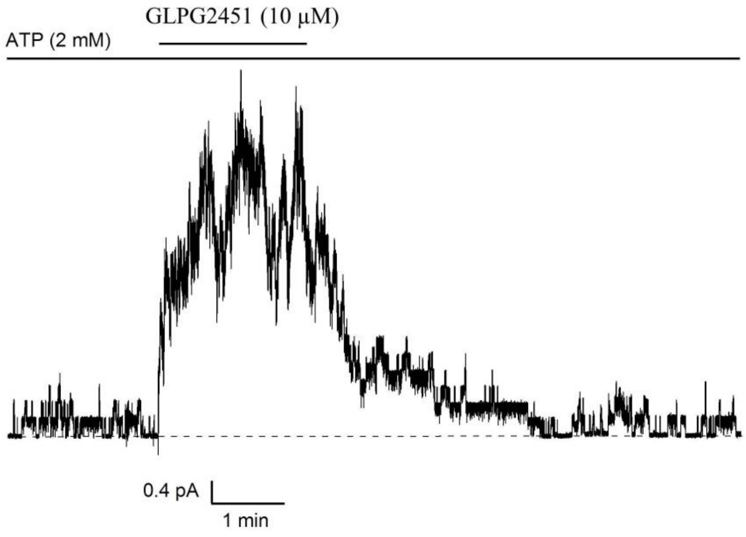

Supplementary Figure 3.

Example of the time course of current activation and potentiation by GLPG2451 during a patch clamp experiment on excised patches using CHO cells expressing F508del CFTR.
